# Application of Response Surface Methodology to Improve the Tableting Properties of Poorly Compactable and High-Drug-Loading Canagliflozin Using Nano-Sized Colloidal Silica

**DOI:** 10.3390/pharmaceutics15112552

**Published:** 2023-10-29

**Authors:** Majed Alrobaian, Ahmed Alalaiwe, Ziyad S. Almalki, Mohamed H. Fayed

**Affiliations:** 1 Department of Pharmaceutics and Industrial Pharmacy, College of Pharmacy, Taif University, Taif 21944, Saudi Arabia; majed.alrobaian@tu.edu.sa; 2 Department of Pharmaceutics, College of Pharmacy, Prince Sattam Bin Abdulaziz University, Al-kharj 11942, Saudi Arabia; a.alalaiwe@psau.edu.sa; 3 Department of Clinical Pharmacy, College of Pharmacy, Prince Sattam Bin Abdulaziz University, Al-kharj 11942, Saudi Arabia; z.almalki@psau.edu.sa; 4 Department of Pharmaceutics, College of Pharmacy, University of Hafr Albatin, Hafr Albatin 31991, Saudi Arabia; 5 Department of Pharmaceutics, Faculty of Pharmacy, Fayoum University, Fayoum 63514, Egypt

**Keywords:** tableting of canagliflozin, nano-sized colloidal silica, design of experiment, high drug loading

## Abstract

Designing a robust direct compression (DC) formulation for an active pharmaceutical ingredient (API) with poor flow and compaction properties at a high API load is challenging. This study tackled two challenges: the unfavorable flow characteristics and tableting problems associated with a high-drug-loading canagliflozin (CNG), facilitating high-speed DC tableting. This was accomplished through a single-step dry coating process using hydrophilic nano-sized colloidal silica. A 3^2^ full-factorial experimental design was carried out to optimize the independent process variables, namely, the weight percent of silica nanoparticles (X_1_) and mixing time (X_2_). Flow, bulk density, and compaction properties of CNG–silica blends were investigated, and the optimized blend was subsequently compressed into tablets using the DC technique. A regression analysis exhibited a significant (*p* ≤ 0.05) influence of both X_1_ and X_2_ on the characteristics of CNG with a predominant effect of X_1_. Additionally, robust tablets were produced from the processed powders in comparison with those from the control batch. Furthermore, the produced tablets showed significantly lower tablet ejection forces than those from the control batch, highlighting the lubrication impact of the silica nanoparticles. Interestingly, these tablets displayed improved disintegration time and dissolution rates. In conclusion, a dry coating process using silica nanoparticles presents a chance to address the poor flow and tableting problems of CNG, while minimizing the need for excessive excipients, which is crucial for the effective development of a small-sized tablet and the achievement of a cost-effective manufacturing process.

## 1. Introduction

Canagliflozin (CNG) is an oral anti-diabetic drug that falls under the category of sodium-glucose cotransporter-2 (SGLT-2) inhibitors. Further, CNG has the potential to decrease the absorption of glucose in cancerous breast tissue, which can impede the growth and multiplication of tumor masses [1].

The tableting of canagliflozin (CNG) poses significant challenges because of its low flowability, poor compaction properties, and high drug loading [2]. Additionally, metformin is commonly used in combination with other hypoglycemic drugs, like CNG, to produce a fixed dose formulation, which consequently increases the tablet size and could potentially negatively influence its compressibility [3]. To address these difficulties, the wet granulation method has been employed to create drug-excipient agglomerates that possess suitable flow and compaction characteristics [4]. Nevertheless, wet granulation is a complex procedure with multiple variables, making it challenging to control [2,5,6]. Moreover, it involves time-consuming wetting and drying steps, which can lead to decreased activity of the active components [7]. Issues such as fluctuations in feed through the hopper and increased manufacturing costs have also been identified as drawbacks of this method [7].

The production of oral solid dosage forms (i.e., tablets) can be accomplished through various processing methods, each varying in cost and complexity [2]. The most straightforward and cost-efficient approach is the direct compression process (DC) [6,8]. In contrast to more intricate techniques like dry and wet granulation, DC requires less expensive equipment, facilitates faster processing, and follows a straightforward and efficient process pathway [9]. These factors contribute to DC tableting being the preferred method for tablet manufacturing, especially when high-speed DC tableting is viable. Nevertheless, certain products, especially those with a substantial amount of active pharmaceutical ingredients (APIs), pose challenges when it comes to processing them into tablets through DC. The primary obstacles are the unfavorable flow or compression characteristics exhibited by the APIs and/or excipient blends [10]. Thus, continuous DC tableting exhibits a high degree of sensitivity to the characteristics of the powder, particularly its flowability and bulk density. These attributes have a significant role in determining the quality of the produced tablets. To ensure uniform die filling and produce tablets with sufficient tensile strength, it is imperative for powders to exhibit adequate flow properties and effective packing, respectively. In cases of poor flow, it can lead to detrimental process interruptions, directly influencing product uniformity. Additionally, the bulk properties of the powder and its compressibility also have a direct impact on both the pre-compaction and compaction stages of the process [11,12].

Dry coating is a technique where the surface of larger host particles is modified by applying a coating of much smaller guest particles, usually in the nanoscale range [13]. This process smooths out the roughness of the larger particles’ surfaces and creates a physical barrier between them [14]. Previous studies have investigated the use of dry coating as a technique to reduce interparticle cohesion [11,15]. This method has shown promising potential to facilitate DC tableting by improving the powder properties, including flow, bulk density, and compaction [11,12,16]. However, designing a robust DC formulation for an API with poor flow and compaction properties at a high API load (i.e., canagliflozin) is challenging [2]. In addition, increasing the drug-loading level can substantially diminish the size of the solid dosage form (i.e., tablet), leading to a consequent reduction in manufacturing expenses [17].

Therefore, the main objective of this study was to examine the feasibility of the DC of CNG powder, by improving flowability and compaction properties, which was prepared with a single-step process using nano-sized colloidal silica. The additional challenge was to design a robust DC formulation for CNG at a high loading level. Cohesive CNG powders were co-processed using hydrophilic colloidal silica nanoparticles. Blend flowability, the DC tableting of processed CNG, and the disintegration and dissolution performances of the corresponding tablets were investigated. 

## 2. Materials and Methods

### 2.1. Materials

Canagliflozin and nano-sized hydrophilic fumed silica “Aerosil 200^®^” were supplied by JPI Ltd. Co. (Al-kharj, Saudi Arabia). Polyvinylpyrrolidone (Kollidon^®^ 25) was procured from BASF Co. (Ludwigshafen, Germany). Croscarmellose sodium (Vivasol^®^) was purchased from JRS pharma (Rosenberg, Germany). All the other chemicals were of analytical grade. 

### 2.2. Experimental Design 

A series of preliminary experiments were conducted prior to generating the design aimed at identifying suitable ranges for the variables to yield powder blends with desirable properties. A randomized full-factorial design of 3^2^ runs was executed using Design-Expert software (version 12, State-ease, Inc., Minneapolis, MN, USA) to assess the impact of two independent variables, the weight percent of silica nanoparticles (X_1_) and mixing time (X_2_) of the tableting process of CNG. The chosen levels of these variables, low (−1), medium (0), and high (+1), are displayed in Table 1. The complete matrix of the design, as provided by the software, is shown in Table 2. The response variables investigated included angle of repose (Y_1_), bulk density (Y_2_), and tensile strength (Y_3_). Each measurement was conducted three times, and the data were presented as the mean ± standard deviation (SD).

The Design-Expert software facilitated the creation of linear, interaction, and quadratic terms for all the response variables. The selection of the most appropriate model was based on a comparison of statistical indicators supplied by the software. Furthermore, a multiple regression analysis was conducted at a significance level of 95% to identify the significant impacts of the investigated variables on the regression coefficients of the responses. For the optimization phase, the correlation between the investigated variables and the measured responses was established and modeled using (Equation (1)).

(1) $$Y=b_{0}+b_{1}X_{1}+b_{2}X_{2}+b_{12}X_{1}X_{2}+b_{11}X_{1}{}^{2}+b_{22}X_{2}{}^{2}$$

In the given equations, Y represents the measured response, b_0_ stands for the intercept, X_1_ and X_2_ denote the coded levels of the investigated variables, b_1_ and b_2_ are the regression coefficients, b_12_ refers to the interaction term, and b_11_ and b_22_ represent the quadratic coefficients. The verification of the design’s accuracy was accomplished using Equation (2). A relative error (RE) of less than 5% was anticipated to validate the precision of the design [18]. The formula for relative error is as follows:

(2) $$\mathrm{RE}=\left(\;\frac{\mathrm{Predicted}\;\mathrm{value}-\mathrm{Experimental}\;\mathrm{value}\;}{\mathrm{Predicted}\;\mathrm{value}}\right)\times 100$$

### 2.3. Preparing Powder Blends 

A high-shear mixer with a capacity of 2 L (BOSCH Packaging Technology, Schopfheim, Germany) was employed to create a powder mixture. The blending process involved loading a combination of CNG and silica nanoparticle powders into the mixer bowl. The specific arrangement for the blending process was determined according to the details provided in Table 2. Throughout all the experiments, a consistent set of operating conditions was maintained, including a 65% filling volume and an impeller speed of 250 rpm, determined according to the previous testing.

### 2.4. Evaluation of Blends

#### 2.4.1. Flowability 

The flow properties of the blends were assessed three times utilizing the angle of repose (AoR) technique. The powder was introduced into a constant-height dry conical funnel and allowed to flow onto a circular plate, forming a well-proportioned cone. The diameter (D) of the cone’s base was gauged, and the AoR (°) was computed using Equation (3) [19].

(3) $$\tan\left(\alpha \right)=\frac{2H}{D}$$

#### 2.4.2. Bulk Density 

The USP method was employed to determine the bulk density (ρb) in three separate trials [19]. A quantity of 15 g of the powder mixture (m) was gently poured, avoiding compression, into a 50 mL graduated cylinder. The volume (Vb) occupied by the blend was then measured, and the bulk density (ρb) was computed using Equation (4).

(4) $$\rho b=\frac{m}{\mathrm{Vb}}$$

#### 2.4.3. Powder Compaction 

The powder mixtures were compressed into tablets of 119 mg each, using a single-punch machine (Korsch Pressen, Berlin, Germany) equipped with round, flat-faced punches measuring 7.0 mm in diameter at a 30 rpm turret speed and a compression force of 10 kN. Subsequently, the thickness (t) and diameter (d) of the resulting tablets were measured using a digital caliper. To determine the breaking force (F) of the tablets, a hardness tester (Erweka, Heusenstamm, Germany) was utilized. The tablets’ tensile strength (σ) was computed using Equation (5).

(5) $$\sigma \;=\frac{2F}{\pi \mathrm{td}}$$

### 2.5. Tableting Process 

Table 3 displays the exact formulation of the canagliflozin tablets, with a batch size of 300 g. The optimized mixture of CNG and silica was blended with 10%*w*/*w* of PVP K25 and 5%*w*/*w* of CCS using a high-shear mixer. The mixer operated at an impeller speed of 250 rpm for a duration of 2 min. Subsequently, the resulting blend was directly compressed into 119 mg tablets using a compaction force of 10 kN, employing a rotary tablet press (RoTap, Kg Pharma, Berlin, Germany). The tablet press was set up to produce 4 tablets per cycle using a 7.0 mm flat-tablet tooling. The control batch, which did not contain silica nanoparticles, was prepared following the same procedure. 

### 2.6. Evaluation of the Prepared Tablets

#### 2.6.1. Weight Variation

The weight variation of the prepared tablets was assessed with the USP standard procedure [19] for 20 tablets randomly selected from each batch.

#### 2.6.2. Tensile Strength and Friability 

The measurement of tablet tensile strength was conducted in accordance with the procedure outlined in the earlier Section 2.4.3. In addition, tablet friability was assessed following the guidelines of the USP standard protocol [19]. This involved placing 20 tablets in a friabilator (ERWEKA, Heusenstamm, Germany) and rotating them at a speed of 25 rpm for a duration of 4 min. The percentage weight loss following the tumbling process was then computed using Equation (6).

(6) $$\mathrm{Friability}\;\left(\%\right)=\left(\frac{W_{1}-W_{2}}{W_{1}}\right)\times 100$$

where W_1_ represents the initial weight of the tablets before undergoing the tumbling process and W_2_ represents the weight of the tablets after they were rotated in the friabilator.

#### 2.6.3. Disintegration of Tablets

The in vitro disintegration assessment was performed on a set of six tablets chosen at random. This was accomplished utilizing a disintegration tester (Erweka, ZT4, Heusenstamm, Germany) within 800 mL of deionized water adjusted to 37 ± 0.5 °C. The disintegration time (DT) for each tablet was observed and noted in minutes. The DT was determined as the moment when the entire solid content had completely passed through the wire mesh of the disintegration apparatus. The outcomes are displayed as the average value accompanied by the SD.

#### 2.6.4. In Vitro Dissolution

The dissolution study was performed using a dissolution apparatus type II (ERWEKA, Heusenstamm, Germany). The dissolution medium comprised 900 mL of a phosphate buffer solution with a pH of 6.8, kept at 37 ± 0.5 °C, with the paddle operating at a speed of 75 rpm. At different time intervals (5, 10, 15, 20, and 30 min), samples were extracted from the medium. To determine the content of CNG, a UV spectrophotometer (Shimadzu, UV-1800, Kyoto, Japan), set at a wavelength of maximum absorption (λ_max_) of 290 nm, was employed [17]. The percentage of CNG released was computed using a previously established standard calibration curve.

## 3. Results and Discussion

### 3.1. Results of the Model Fitting

**Figure 1:**
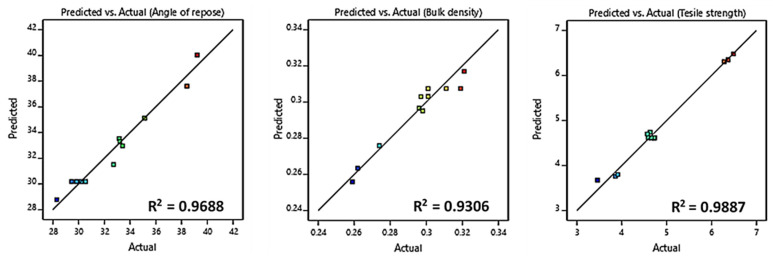
Results of the model fitting with scaled actual versus predicted responses.

Table 4 exhibits the results of the data fitting of the suggested models. The Design-Expert software had the capability to generate mathematical polynomial models encompassing various types, such as linear, two-factor interaction, quadratic, and cubic, which established relationships between the variables and responses. For each response, a value of *p* < 0.05 demonstrated that the terms within the model accurately represented the behavior of the response function. Additionally, the adjusted R^2^ of the chosen model exhibited satisfactory agreement with the predicted R^2^. Determination coefficients (R^2^) exceeding 0.8011 affirmed the appropriateness and accuracy of the suggested models. Furthermore, plots with points close to the regression line indicated good models (Figure 1). 

### 3.2. Influence of Variables on Powder Flow

The sufficient, predictable, and consistent powder flows into a tablet die is essential to maintain uniform tablet weight and drug content [11]. As presented in Table 5, the AoR of the powder blend varied from 39.21 ± 0.223 to 28.31 ± 0.253°. It was evident that the blending of cohesive powder with colloidal silica nanoparticles improved the flow properties. Equation (7) expresses the impact of the independent variables on the powder flow.

(7) $$\mathrm{Angle}\;\mathrm{of}\;\mathrm{repose}\;\left(\mathrm{degree}\right)=30.19-2.16{\;X}_{1}-1.37{\;X}_{2}+1.08{\;X}_{1}X_{2}+5.26{\;X}_{1}{}^{2}-0.043{\;X}_{2}{}^{2}$$

**Figure 2:**
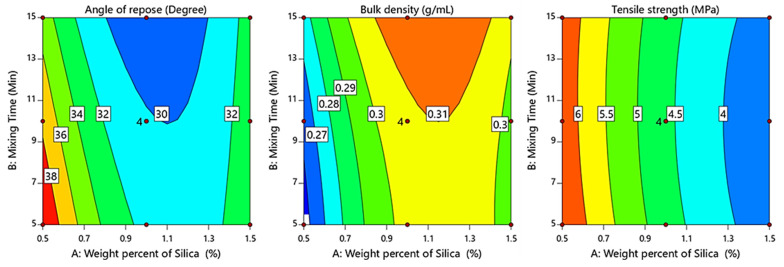
Response contour plots showing the influence of the weight percent of nano-sized colloidal silica (X_1_) and mixing time (X_2_) on AoR (Y_1_), bulk density (Y_2_), and tensile strength (Y_3_).

An ANOVA shown in Table 6 demonstrated that the weight percent of the silica nanoparticles and mixing time had significant effects on powder flow (*p* < 0.0007 for the weight percent of the silica nanoparticles and *p* < 0.0065 for the mixing time). Moreover, the weight percent of the silica nanoparticles had a pronounced effect, followed by the mixing time, based on the values of the sum of squares (28.08 for the weight percent of nano-sized colloidal silica and 11.29 for the mixing time). Further, the interaction term (X_1_X_2_) had a significant impact (*p* < 0.039) on powder flow (i.e., AoR). On the other hand, the negative sign of the regression coefficients in Equation (7) (−2.16 for the weight percent of nano-sized colloidal silica and −1.37 for the mixing time) demonstrated that both variables had positive impacts on decreasing the angle-of-repose value. However, by increasing the weight percent of nano-sized colloidal silica over 1.0%*w*/*w*, the AoR of the powder blend significantly increased from 28.31 ± 0.253° to 33.41 ± 0.244°. This demonstrated that powder blends containing 1.5%*w*/*w* of silica (Runs 7–9) showed a greater AoR value than those containing only 1.0% of silica (Run 4–6). Figure 2 shows that the AoR reached its minimum value at a medium weight percent of nano-sized colloidal silica with a simultaneous increase in the levels of both variables with a pronounced effect of the weight percent of nano-sized colloidal silica. This indicated that a powder blend with 1.0% silica achieved the best flowability. The flow improvements could be attributed to the combined impacts of different mechanisms. When the particles of nano-sized silica were suited between cohesive host particles, they increased the distance between the host particles, leading to a decrease in the contact area. Further, the movement of these mobile silica particles between two host particles could diminish friction forces and enhance the ability of the material to flow smoothly. Additional mechanisms may have involved alterations in surface energy and changes in electrostatic charging due to the presence of a silica coating [6,12]. Conversely, when the concentration of nano-sized colloidal silica was high (i.e., 1.5%), it resulted in colloidal silica particles not only covering the surface of the CNG particles, but also aggregating into various-sized clusters, leading to the formation of a multi-layered coating. This, in turn, resulted in the creation of a larger contact surface area, hindering the rolling motion. Consequently, this impeded the mobility of CNG particles and subsequently compromised the flow characteristics of the mixtures [20,21]. It was reported that there exists a distinct correlation between the extent and uniformity of the coverage of nano-sized colloidal silica on the surface of powder particles and an improvement in flow facilitated by the glidant [21].

**Table 5:** Characterization of prepared CNG–silica blends.

Run	Angle of Repose (°)	Bulk Density (g/mL)	Tensile Strength (MPa)
1	39.21 ± 0.223	0.259 ± 0.033	6.49 ± 0.228
2	38.42 ± 0.337	0.262 ± 0.051	6.28 ± 0.336
3	35.13 ± 0.188	0.274 ± 0.113	6.37 ± 0.265
4	32.71 ± 0.164	0.297 ± 0.025	4.63 ± 0.335
5	29.84 ± 0.198	0.301 ± 0.083	4.72 ± 0.287
6	28.31 ± 0.253	0.321 ± 0.036	4.57 ± 0.235
7	33.16 ± 0.266	0.298 ± 0.081	3.86 ± 0.361
8	33.21 ± 0.321	0.296 ± 0.061	3.46 ± 0.384
9	33.41 ± 0.244	0.301 ± 0.021	3.91 ± 0.236

### 3.3. Influence of Variables on Bulk Density of Powder Blend

As shown in Table 5, the bulk density of the powder blend ranged from 0.259 ± 0.033 to 0.321 ± 0.036 g/mL. It was evident that the addition of nano-sized colloidal silica improved the bulk density of the cohesive powder. These results were in agreement with previously reported studies [11]. Equation (8) expresses the effect of the investigated variables on the bulk density of the powder blend.

(8) $$\mathrm{Bulk}\;\mathrm{density}\;\left(g/\mathrm{mL}\right)=0.3075+0.167{\;X}_{1}+0.007{\;X}_{2}-0.003{\;X}_{1}X_{2}-0.027{\;X}_{1}{}^{2}+0.002{\;X}_{2}{}^{2}$$

An ANOVA, shown in Table 6, revealed that the weight percent of the silica nanoparticles had a significant effect on the bulk density (*p* = 0.0013 for the weight percent of nano-sized colloidal silica and *p* = 0.054 for the mixing time). On the other hand, the positive sign of the regression coefficients in Equation (8) revealed that the significant term had a positive effect on the bulk density (+0.0167 for the weight percent of the silica nanoparticles), showing that increasing the weight percent of nano-sized colloidal silica resulted in a higher bulk density of the powder blend. Figure 2 shows that a medium level of silica and long mixing time resulted in a higher bulk density. However, the contour plot demonstrated that the weight percent of nano-sized colloidal silica had a predominant impact on the bulk density of the powder blend in a positive direction. These observations were attributed to decreased cohesion among the drug particles as well as the de-agglomeration of the drug particles [12]. Furthermore, the improvement in bulk density was more significant in the case of the high-drug-loaded blends [14,22]. 

### 3.4. Influence of Variables on Blend Compaction

As shown in Table 6, the tensile strength of the powder blend ranged from 6.49 ± 0.228 to 3.46 ± 0.384 MPa. Capping, breaking, or other tablet defects were not observed. It was evident that the addition of nano-sized colloidal silica provided the tablets with acceptable tensile strength. It has been reported that the acceptable tensile strength of powder blends for use in DC tableting was 2 MPa [23,24]. These results could be explained by the presence of the hard, nano-sized colloidal silica, which introduced an extra bonding area between the interacting, softer particles. This led to a more robust interparticle strength. Furthermore, the bonding strength between the CNG powder and silica proved to be stronger than the bonding strength between CNG powders. These findings aligned with results from another separate research group [6]. In addition, the presence of nano-sized colloidal silica facilitated the rearrangement of powder particles during the compression of powder [25]. Equation (9) expresses the effect of the investigated variables on powder compaction.

(9) $$\mathrm{Tensile}\;\mathrm{strength}\;\left(\mathrm{MPa}\right)=4.61-1.32{\;X}_{1}-0.0217{\;X}_{2}+0.042{\;X}_{1}X_{2}+0.376{\;X}_{1}{}^{2}-0.106{\;X}_{2}{}^{2}$$

An ANOVA, shown in Table 6, demonstrated that the weight percent of nano-sized colloidal silica had significant effects on the compaction of the powder blend (*p* < 0.0001 for the weight percent of nano-sized colloidal silica and *p* = 0.726 for the mixing time). Moreover, the interaction term (X_1_X_2_) was found to have a statistically non-significant effect (*p* > 0.05) on blend compaction. On the other hand, the negative sign of the regression coefficients in Equation (9) (−1.32 for the weight percent of nano-sized colloidal silica and −0.0217 for the mixing time) demonstrated that the significant term had a negative effect on the tensile strength of the powder blend. Therefore, the reduction of tablet tensile strength was more predominant after the addition of a high amount of nano-sized colloidal silica, as shown in the contour plot of Figure 2. This could be explained by the silica particles at the bonding interfaces, which diminished the bonding area between the CNG particles. Furthermore, the bonding area between the nano-sized colloidal silica particles was limited, as they did not experience plastic deformation during compression. Nano-sized colloidal silica primarily undergoes elastic deformation, which does not lead to permanent bonding between CNG particles [23]. These findings aligned with similar results reported by another research group [23]. 

### 3.5. Optimization of Independent Variables

**Figure 3:**
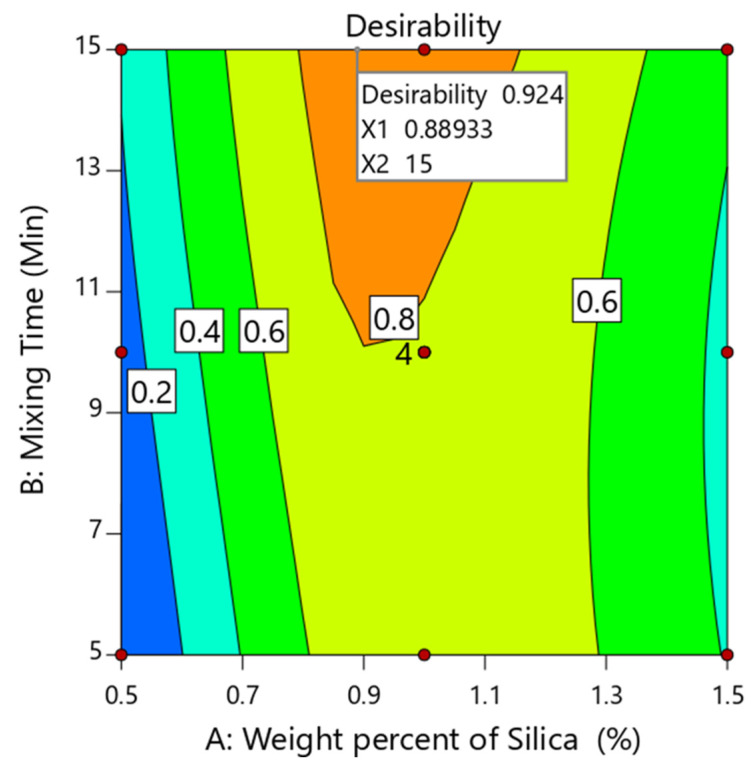
Desirability plot for optimization of investigated variables.

The primary objective of the optimization phase was to refine the independent variables to achieve a product with specific desired attributes [26]. Numerical optimization was performed for the optimization of the investigated variables, wherein targets were set for each selected response. As outlined in Table 7, the desired outcomes were the minimization of AoR (Y_1_), the maximization of bulk density (Y_2_), and a tensile strength (Y_3_) of 5 MPa. Utilizing these criteria, a desirability plot was generated (Figure 3). The optimal conditions were identified as a weight percent of nano-sized colloidal silica of 0.889%*w*/*w* and a mixing time of 15 min. As shown in Table 8, the experimental values for the selected responses, which included AoR, bulk density, and tensile strength, were recorded as 29.91 ± 0.225°, 0.308 ± 0.124 g/mL, and 5.06 ± 0.662 MPa, respectively. Furthermore, the RE was determined by comparing the predicted with the experimental values of the optimized CNG–silica blend. The results indicated that the RE fell within an acceptable value (±5%), demonstrating the validity of the design (Table 8).

### 3.6. Tableting of Optimized CNG–Silica Blend

Assessing the initial appropriateness of the optimized blend for high-speed DC tableting is crucial for identifying whether any adjustments to the formulation or processing are necessary to ensure smooth and efficient processing [27]. According to the recommendations of Sun et al., the optimized blend is likely to be suitable for continuous DC tableting in terms of bulk density and flowability [24]. Table 9 displays weight variation data for tablets produced from the optimized and control blend. It was noted that the tablets produced from the control blend displayed significant weight variation (an RSD of more than 2.0%) compared with those produced using the optimized blend (an RSD of 0.92). These results suggested that the optimized blend exhibited excellent flow characteristics, leading to the production of tablets with a consistent weight. 

The act of ejecting tablets during high-speed DC tableting is important to avoid tablet deterioration, such as lamination, capping, and the powder adhering to the die or the punches of the tablet press [6,27]. In Table 9, a significant difference (*p* < 0.05) in ejection force was noted between the tablets produced with the control batch and CNG–silica optimized blend. Additionally, it was observed that the tablets from the optimized blend had a low ejection force, which suggested that the silica nanoparticles could serve a dual purpose of providing flow aid and lubrication effects. Further, the tablets that were prepared had a friability of less than 0.1%, which was considered desirable. This indicated that the tablets had sufficient mechanical strength and were not easily prone to breaking. 

**Figure 4:**
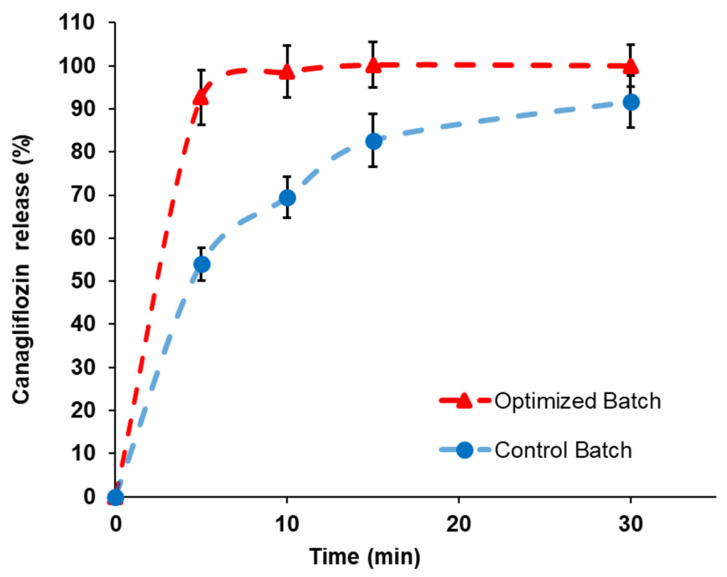
Dissolution profiles of CNG tablets.

Disintegration and dissolution performances are of the utmost importance in ensuring the therapeutic efficacy of immediate-release tablets. Quick disintegration is particularly essential for most immediate-release tablets because it leads to faster dissolution. As the tablet fractures into smaller fragments, the available surface area for dissolution expands, leading to a notably increased dissolution rate [28]. The quicker the drug dissolves, the faster it is absorbed and the higher its bioavailability, leading to immediate and/or stronger effects. As shown in Table 9, there was a significant difference (*p* < 0.05) in the DT between the tablets prepared with the control blend and those prepared with the optimized CNG–silica blend. The DT of the tablets produced with the control blend was 14 ± 0.79 min; in contrast, that of the tablets produced with the optimized CNG–silica blend was 6 ± 0.44 min. The substantially shorter DT of the tablets produced with the optimized CNG–silica blend indicated that the silica nanoparticles facilitated the de-agglomeration of the drug particles [6]. Interestingly, the silica nanoparticles did not prevent the ingress of water into the produced tablets. The dissolution plot of the produced tablets is presented in Figure 4. It is worth noting that the tablets produced with the optimized CNG–silica blend exhibited faster dissolution rates compared with those of the control blend. It was observed that the tablets from the optimized blend released over 90% of the CNG within 5 min, which was consistent with the disintegration performance. The enhanced dissolution of CNG could be attributed to increased wettability and a reduction in the strength of drug agglomeration, leading to enhanced dispersion within the dissolution medium [22,29]. These data demonstrated that powder cohesion had a significant effect on the DT and dissolution rate. 

## 4. Conclusions

The effects of nano-sized colloidal silica and mixing time on the high-drug-loaded blend and tablet properties were thoroughly examined for the cohesive and poorly compactable API “canagliflozin”. The processed drug powders showed the effective modification of interparticle interactions as proved by significant alterations in flowability, bulk density, and compaction properties. Moreover, robust tablets could be prepared with the processed powders. Interestingly, the produced tablets showed significantly lower tablet ejection forces, highlighting the lubrication influence of the silica nanoparticles. Additionally, these tablets displayed improved DT and dissolution rates. Therefore, this research provides evidence for the hypothesis that it is possible to create a one-step method for generating a powdered mixture with integrated qualities suitable for the DC tableting of cohesive and poorly compactable APIs. This could serve as a substitute for the conventional multi-stage granulation procedures. Further, increasing the drug-loading level can substantially reduce the size of the tablets, making them easier to swallow as well as consequently reducing the manufacturing cost.

## Figures and Tables

**Table 1:** Investigated parameters and levels.

Coded Levels	Weight Percent of Nano-Sized Colloidal Silica (%*w*/*w*)	Mixing Time (min)
−1	0.5	5
0	1	10
+1	1.5	15

Low, medium, and high levels are denoted by –1, 0, and +1, respectively.

**Table 2:** A full matrix of the generated design.

Run	Weight Percent of Silica Nanoparticles (%*w*/*w*)	Mixing Time (min)
1	0.5	5
2	0.5	10
3	0.5	15
4	1	5
5	1	10
6	1	15
7	1.5	5
8	1.5	10
9	1.5	15

**Table 3:** Formulation of canagliflozin tablets (batch of 300 g).

Ingredients	%*w*/*w*	Quantity (g)
Canagliflozin	84.12	252.36
Nano-sized colloidal silica	0.88	2.64
Polyvinylpyrrolidone (PVP K25)	10	30
Croscarmellose sodium (CCS)	5	15

**Table 4:** Data fitting of the model.

Responses	Suggested Model	*p*-Value	R^2^	Adjusted R^2^	Predicted R^2^	Adequate Precision
Y_1_: Angle of repose (°)	Quadratic	0.0002	0.9688	0.9429	0.7421	19.308
Y_2_: Bulk density (g/mL)	Quadratic	0.002	0.9306	0.8727	0.7343	12.047
Y_3_: Tensile strength (MPa)	Quadratic	<0.0001	0.9887	0.9793	0.9211	27.446

**Table 6:** Regression analysis (ANOVA) of selected responses.

Variables	Coefficient Estimate	Sum of Squares	Standard Error	F-Value	*p*-Value	95% CI Low	95% CI High
Y_1_: Angle of repose (Quadratic model)							
Intercept	30.19	-	0.3761	-	-	29.27	31.11
X_1_	−2.16	28.08	0.3364	41.36	0.0007	−2.99	−1.34
X_2_	−1.37	11.29	0.3364	16.63	0.0065	−2.19	−0.548
X_1_X_2_	1.08	4.69	0.4120	6.90	0.0392	0.074	2.09
X_1_^2^	5.26	73.82	0.5046	108.72	<0.0001	4.03	6.50
X_2_^2^	−0.0437	0.0051	0.5046	0.0075	0.9337	−1.28	1.19
Y_2_: Bulk density (Quadratic model)							
Intercept	0.3075	-	0.0033	-	-	0.2995	0.3155
X_1_	0.0167	0.0017	0.0029	32.33	0.0013	0.0095	0.0238
X_2_	0.007	0.0003	0.0029	5.70	0.0542	−0.0002	0.0142
X_1_X_2_	−0.003	0.000	0.0036	0.6983	0.4354	−0.0118	0.0058
X_1_^2^	−0.0275	0.002	0.0044	39.12	0.0008	−0.0383	−0.0167
X_2_^2^	0.0025	0.000	0.0044	0.3233	0.5903	−0.0383	−0.0133
Y_3_: Tensile strength (Quadratic model)							
Intercept	4.61	-	0.066	-	-	4.45	4.78
X_1_	−1.32	10.43	0.059	498.47	<0.0001	−1.46	−1.17
X_2_	−0.0217	0.0028	0.059	0.1346	0.7263	−0.1662	0.1228
X_1_X_2_	0.0425	0.0072	0.0723	0.3454	0.5782	−0.1345	0.2195
X_1_^2^	0.3763	0.3775	0.0886	18.05	0.0054	0.1595	0.5930
X_2_^2^	0.1063	0.0301	0.0886	1.44	0.2755	−0.1105	0.3230

X_1_ and X_2_ are the weight percent of nano-sized colloidal silica and mixing time, respectively; X_1_X_2_ is the interaction effect.

**Table 7:** Optimization of investigated variables.

Variables	Target	Range	Weight	Importance Co-Efficient
**Input**				
Silica weight percent	In range	0.5–1.5%	1	NA *
Mixing time	In range	5–15 min	1	NA
**Output**				
Angle of repose	Minimize	28.31–39.21°	1	+++
Bulk density	Maximize	0.259–0.321 g/mL	1	+++
Tensile strength	5	3.46–4.99 MPa	1	+++

* Not Applicable.

**Table 8:** The optimal condition values and the predicted versus experimental values for the selected responses of the optimized CNG–silica blend.

Variables	Value		
Weight percent of nano-sized silica	0.889%*w*/*w*		
Mixing time	15 min		
Overall desirability = 0.924			
**Responses**	**Predicted Values**	**Experimental Values**	**Relative Error (%)**
Angle of repose (°)	29.27	29.91 ± 0.225	−2.18
Bulk density (g/mL)	0.312	0.308 ± 0.124	4.04
Tensile strength (MPa)	4.99	5.06 ± 0.662	−1.40

**Table 9:** Properties of produced tablets produced with optimized CNG–silica blend.

Properties	Optimized CNG–Silica Blend	Control Blend **
RSD of weight variation (%)	0.92	2.54
Ejection force (MPa)	104 ± 3 *	169 ± 5
Friability (%)	0.02 ± 0.16	0.06 ± 0.14
Tensile strength (MPa)	5.06 ± 0.662	6.72 ± 0.81
Disintegration time (min)	6 ± 0.44 *	14 ± 0.79

* Significant difference at *p* < 0.05; ** blend without silica was denoted as a control batch.

## Data Availability

The datasets used and/or analyzed during the current study are available from the corresponding author on reasonable request.
